# Rapid establishment of a national surveillance of COVID-19 hospitalizations in Belgium

**DOI:** 10.1186/s13690-020-00505-z

**Published:** 2020-11-18

**Authors:** Nina Van Goethem, Aline Vilain, Chloé Wyndham-Thomas, Jessika Deblonde, Nathalie Bossuyt, Tinne Lernout, Javiera Rebolledo Gonzalez, Sophie Quoilin, Vincent Melis, Dominique Van Beckhoven

**Affiliations:** 1 Scientific Directorate of Epidemiology and public health, Sciensano, J. Wytsmanstraat 14, 1050 Brussels, Belgium; 2 Department of Epidemiology and Biostatistics, Institut de recherche expérimentale et clinique, Faculty of Public Health, Université catholique de Louvain, Clos Chapelle-aux-champs 30, 1200 Woluwe-Saint-Lambert, Belgium; 3 Directorate Healthcare, Federal Public Service (FPS) Health, Food Chain Safety and Environment, Brussels, Belgium

**Keywords:** COVID-19, Hospital-based surveillance, Hospital capacity, Hospital outcome

## Abstract

**Background:**

In response to the COVID-19 epidemic, caused by a novel coronavirus, it was of great importance to rapidly collect as much accurate information as possible in order to characterize the public health threat and support the health authorities in its management. Hospital-based surveillance is paramount to monitor the severity of a disease in the population.

**Methods:**

Two separate surveillance systems, a Surge Capacity survey and a Clinical survey, were set up to collect complementary data on COVID-19 from Belgium’s hospitals. The Surge Capacity survey collects aggregated data to monitor the hospital capacity through occupancy rates of beds and medical devices, and to follow a set of key epidemiological indicators over time. Participation is mandatory and the daily data collection includes prevalence and incidence figures on the number of COVID-19 patients in the hospital. The Clinical survey is strongly recommended by health authorities, focusses on specific patient characteristics and relies on individual patient data provided by the hospitals at admission and discharge.

**Conclusions:**

This national double-level hospital surveillance was implemented very rapidly after the first COVID-19 patients were hospitalized and revealed to be crucial to monitor hospital capacity over time and to better understand the disease in terms of risk groups and outcomes. The two approaches are complementary and serve different needs.

**Supplementary Information:**

The online version contains supplementary material available at 10.1186/s13690-020-00505-z.

## Background

At the end of December 2019, the Chinese public health authorities reported a cluster of cases of pneumonia in Wuhan City, Hubei province, China. The causative agent was identified as a novel coronavirus called severe acute respiratory syndrome coronavirus 2 (SARS-CoV-2). The related disease is referred to as coronavirus disease 2019 (COVID-19). The initial outbreak in Wuhan spread rapidly, affecting other parts of China first and then an increasing number of countries, resulting in a pandemic [1]. The first European country to be severely hit by the epidemic was Italy at the end of February, followed by the other European countries a few weeks later [2, 3].

In Belgium, health authorities reported the first infection on the 4th of February 2020 in an asymptomatic Belgian citizen repatriated from Wuhan [4]. From March onwards, the first symptomatic cases were reported and numbers quickly increased. The outbreak was initially related to the return of holidaymakers, principally from Italy, and was rapidly followed by local- and community-based transmission throughout the country [5].

In the event of a public health threat measures to reduce the risk to the Belgian population are decided by the Risk Management Group (RMG) [6], composed of representatives of the Belgium’s health authorities and coordinated by the National Focal Point for the International Health Regulations of the federal public health service. In order to prepare for a potential surge in COVID-19 patients needing acute and intensive care in hospitals, a series of measures related to COVID-19 management in hospitals was decided as from the 2nd of March 2020 and resulted in the creation of the Hospital & Transport Surge Capacity Plan (HTSC plan) and of the Hospital & Transport Surge Capacity Committee (HTSC committee). The purpose of the HTSC committee is to publish guidelines and recommendations for the hospitals during the crisis, to monitor the COVID-19 activity within hospitals, and to facilitate transfer of COVID-19 patients between hospitals. To monitor the hospital capacity with regards to COVID-19 activities, a surveillance tool, the Surge Capacity (SC) survey, was commissioned to and developed by the Belgian Scientific Institute of Health, Sciensano, given the statutory framework [7]. The survey was designed to capture in real-time the occupancy rate of beds and medical devices within Belgian general hospitals by COVID-19 patients.

In parallel, Sciensano was commissioned by the RMG to collect and process individual health data from hospitalized patients with a confirmed COVID-19 infection. Indeed, with the paucity of background data on COVID-19, inherent to its recent emergence, and as recommended by the ECDC [8], implementing a hospital-based surveillance to identify risk groups for severe disease, measure impact and inform policy makers for their decisions on mitigation measures was indispensable. The Clinical survey set-up for this purpose was designed to measure risk factors and outcomes of COVID-19 hospitalized patients.

Thus two complementary hospital-based surveys were set-up promptly after the beginning of the Belgium’s COVID-19 epidemic. The objective of this paper is to describe the specific methodology of both surveillance systems, discuss their implementation and the methodological choices that were made along the way, and provide insight with regards to the characteristics of the data obtained, the participation rate, and the reporting process.

## Methodology

### Surveillance objectives

The primary objective of the SC survey is to monitor the occupancy rate of beds and medical devices within Belgian general hospitals by COVID-19 patients. Secondly, additional data and indicators obtained through the SC survey, such as the number of new confirmed and possible COVID-19 hospitalizations, discharges and deaths, are to be used for epidemiological purposes to follow trends over time.

The specific objectives of the Clinical survey are to study: (a) demographic and comorbidity profiles of the COVID-19 patients requiring hospitalization; (b) risk factors for Intensive Care Unit (ICU) admission and mortality among hospitalized patients; (c) lengths of stay in hospital, in ICU and on extracorporeal membrane oxygenation (ECMO); (d) biological status and evolution of hospitalized patients with COVID-19 based on selected laboratory measures; and (e) the use of specific medications for COVID-19.

### Surveillance outline

The SC survey has been operational since the 10th of March 2020. A total of 103 general hospitals partake in the survey and their participation is mandatory (Royal Decree of 30.04.2020 [9]). This list includes all general hospitals (including university hospitals) in Belgium, and both hospitals managed by a public authority and privately run are represented. The surveillance does not cover psychiatric hospitals or specialist hospitals (e.g. exclusively providing geriatric, revalidation or palliative care) [10]. Each participating hospital sends their data on a daily basis and before 11 o’clock to Sciensano via a secured online questionnaire. To facilitate participation, reminder emails are sent to hospital contact persons each day at 8 AM. The reminder email contains the link to the survey, and highlights any changes to the survey, as well as a document with Frequently Asked Questions (FAQs).

The collection of individual data of patients hospitalized with confirmed COVID-19 through the Clinical survey was implemented on the 14th of March 2020 using a secured online questionnaire to report all COVID-19 patients hospitalized since the beginning of the epidemic. It comprises two sections: one to fill in after admission of the patient and one to fill in after discharge. Participation to this surveillance is strongly recommended for all hospitals caring for COVID-19 patients, but not mandatory.

Both questionnaires were created using LimeSurvey (GmbH, Hamburg, Germany. URL http://www.limesurvey.org), an open source survey tool. Hospitals can contact Sciensano with questions, feedback, and remarks regarding the surveys.

### Study population

The SC survey collects data on both confirmed and possible COVID-19 patients who are hospitalized in one of the 103 general hospitals, with the exclusion of outpatients and day hospitalizations.

The studied population of the Clinical survey includes all hospitalized patient with a COVID-19 confirmed infection. All hospitals were invited to participate. Initially, only patients confirmed by reverse transcriptase–polymerase chain reaction (RT-PCR) tests were reported. As of the 3rd of April, the data collection was extended to cases diagnosed by chest computed tomography (CT) scan and rapid antigen tests to align with modifications in the official case definition [11].

### Data collection

The SC survey collects data at an aggregated level per hospital accreditation number. Both prevalence and incidence numbers are obtained through the survey. The following variables are collected as a prevalence number: the number of hospitalized COVID-19 patients in all units of the hospital (including ICU), in the ICU, under invasive ventilation (IV), and under Extra-Corporeal Membrane Oxygenation (ECMO). These numbers reflect the state at the moment of reporting, i.e. a snapshot of the situation. The incidence numbers collected reflect the in- and efflux of patients: the number of new COVID-19 hospital admissions in the last 24 h, the number of COVID-19 discharges in the last 24 h, and the number of COVID-19 deaths in the last 24 h. Both prevalence and incidence numbers are recorded separately for lab-confirmed cases (‘lab-confirmed’ category) on the one hand, and CT-scan-confirmed and possible cases (‘CT-confirmed/suspected’ category) on the other hand.

The SC survey had to be adapted on multiple occasions based on emerging knowledge, management and organizational policies, and modifications of testing criteria [12]. Key changes included the stratification of the number of new COVID-19 hospitalizations according to the origin of the patients, to be able to distinguish the epidemic occurring in nursing homes or other long-term care facilities (LTCF) from the one in the general population. Another major addition was the stratification by SARS-CoV-2 testing indication, in order to differentiate COVID-19-related admissions from other admissions diagnosed with COVID-19 through screening. An overview of the adaptations applied to the SC survey is presented in the Supplementary material (see Additional file 1).

Starting from the 24th of March, the database resulting from the SC survey became the official reference for the epidemiological surveillance of in-hospital deaths resulting from COVID-19. Therefore, individual data collection was added for each confirmed and possible COVID-19 patient who died as a result of a COVID-19 infection, including the date of death, date of birth, sex, postal code of residence, and method of diagnosis.

Individual data collected through the admission questionnaire of the Clinical survey include demographic characteristics (date of birth, sex, ethnicity and postal code), exposure risk, clinical presentation, pre-existing comorbidities, uptake of Angiotensin-Converting Enzyme (ACE) inhibitors and Angiotensin II Receptor Blockers (ARBs), and diagnostic information. Data collected at discharge include severity indicators, selected laboratory data, COVID-19 treatment details, admission in ICU, use of IV and ECMO, and discharge information (alive/deceased). The selection of the variables to be collected at patient’s admission was based on the WHO case report form for COVID-19 [13] supplemented by the input from hospital clinicians. With the evolution of the epidemic, new public health and epidemiological questions arose, triggering adaptation of the variables recorded. In consequence, the questionnaires were modified several times in order to ensure that information remain relevant for clinicians and authorities. For the same purpose, specific questions concerning intensive care were added on the 14th of September. The list of the variables with their date of addition/removal is available in the Supplementary material (see Additional file 2).

### Data cleaning, management and analysis

The data from the SC survey is extracted from the LimeSurvey database in csv format. Errors as reported by the hospitals by email or phone are manually corrected in the SC LimeSurvey database. In addition, checks are performed on the data to detect potential inconsistencies, e.g. number of patients in ICU cannot be larger than the number of patients in the hospital. Furthermore, trend analyses per hospital are used to detect erroneous reporting. For the individual mortality data, double entries are identified based on date of birth, date of death, postal code, and reporting hospital. Data cleaning and analyses are performed using Excel and R software (R Studio version 1.0.153 [14]).

Admission and discharge data from the Clinical survey are first screened for identification and removal of duplicate observations. The two datasets are merged using the hospital patient identification number, or alternatively a combination of date of birth, sex and postal code. Extensive consistency checks are then performed with a specific focus on discrepancies between reported dates. Clinical data are extracted from the LimeSurvey and imported in SAS Enterprise Guide 7.1, software in which data management and analyses are performed.

### Data protection, ethical and privacy considerations

Sciensano is legally entitled for surveillance activities related to public health in Belgium [7]. To ensure the validity of the collected data, the access to both surveys is limited to hospitals through an access code provided only to participating hospitals from April 11th onwards. All answers filled in the online survey in Limesurvey are saved on the central server of Sciensano.

Collection of aggregated data, as for the SC survey, are allowed by the General Data Protection Regulation (GDPR) [15]. Concerning the individual mortality data, although GDPR does not apply to the personal data of deceased persons, the researchers notified the Belgian Data Protection Authority. Collection of individual data, as for the Clinical survey, was authorized by the Belgian Data Protection Authority, as required by the GDPR. The data collection for the Clinical survey is retrospective in nature as Sciensano retrieves data from records of patients hospitalized with COVID-19. As such, the Clinical survey was approved by the ethical committee of Ghent University Hospital (BC-07507). The privacy authorization was modified on the 1st of September to allow the collection of patient’s pseudonimized national registry number in the Clinical survey.

A letter of information for the patient on the Clinical survey, the storage period, the right to examine and correct own data and the contact details of the person to contact to exercise those rights was sent to all data providers. Hospitals also received a letter sent by the HTSC committee explaining the mandate and the purpose of both surveys.

### Data reporting and dissemination of data

Since March 14th, data from the SC survey is reported by Sciensano distinctively to the public and authorities in the *epidemiological bulletin* published on a daily basis. Key indicators are reported in both and an extra chapter is included in the authorities report on ICU bed occupancy. Since March 26th, an extra and more detailed report is produced on a weekly basis, including amongst others findings from the data collected by both the SC survey and the Clinical survey.

### Study outcomes

After an implementation phase of 5 days, a participation rate of 100% was reached on March 15th for the SC survey, and remained very high during the whole period. The mean participation rate between the 15th of March and the 28th of June was equal to 99.9%.

**Fig. 1:**
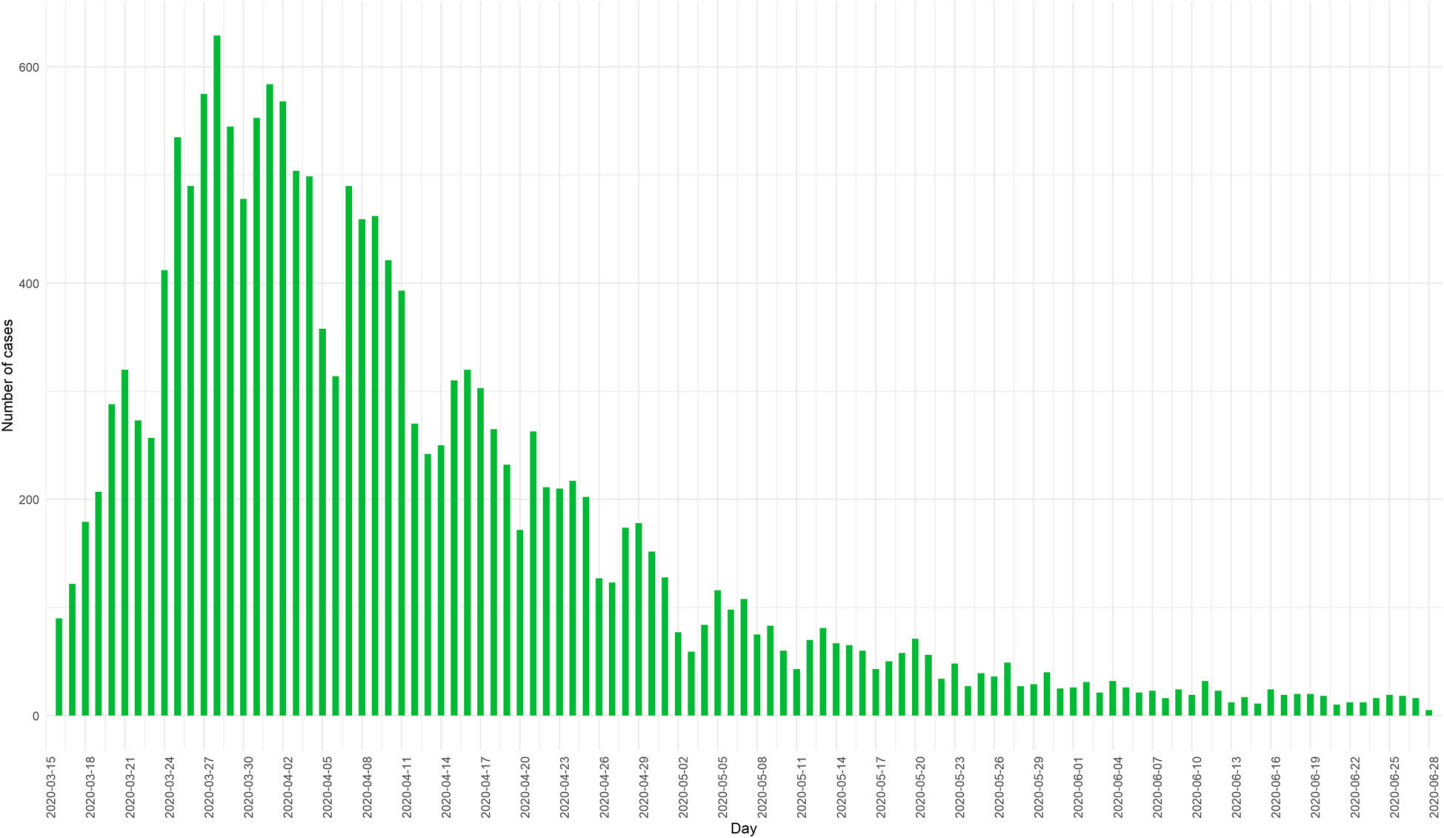
Daily number of new COVID-19-related hospital admissions with a lab-confirmation at the moment of reporting, between the 15th of March 2020 and the 28th of June 2020, Belgium

**Fig. 2:**
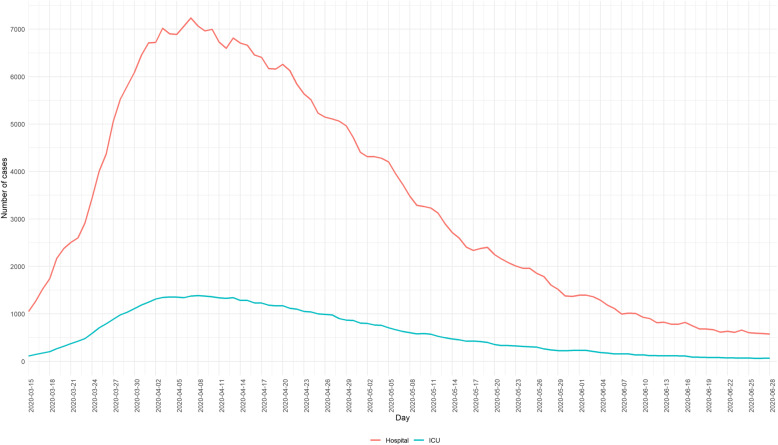
Daily number of confirmed and possible COVID-19 patients in general hospitals and the intensive care units between the 15th of March 2020 and the 28th of June 2020, Belgium

**Fig. 3:**
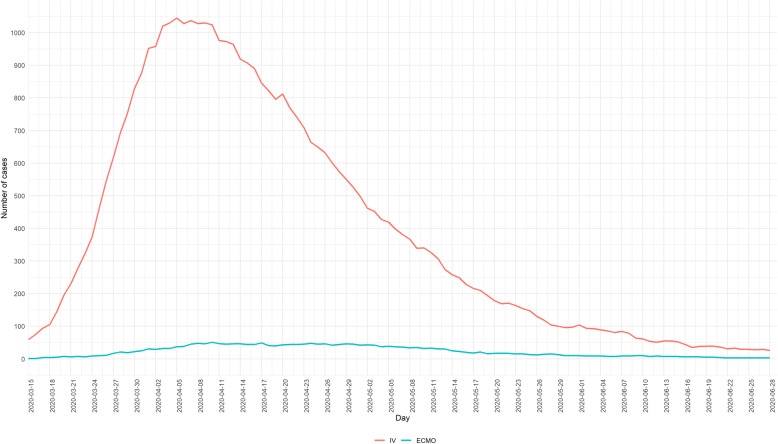
Daily number of confirmed and possible COVID-19 patients in general hospitals on invasive ventilation and extra-corporeal membrane oxygenation between the 15th of March 2020 and the 28th of June 2020, Belgium

Between the 15th of March 2020 and the 28th of June 2020, a total of 17,746 new COVID-19-related hospital admissions with a lab-confirmation available at the moment of reporting have been registered through the SC survey. The evolution over time is presented in Fig. 1. The estimated total number of lab-confirmed COVID-19 patients treated by the general hospitals in Belgium is equal to 21,416 and is calculated as the sum of lab-confirmed COVID-19 discharges and deaths between the 15th of March and the 28th of June, and the number of lab-confirmed COVID-19 patients in the hospital on the 28th of June. The number of occupied beds (Fig. 2) and devices (Fig. 3) by COVID-19 patients has been daily followed. The ICU occupancy rate is reported based on the total number of confirmed and possible COVID-19 patients in ICU as the nominator and the theoretical number of ICU beds reserved for COVID-19 patients (as stipulated in the HTSC plan) as the denominator.

Up to the 28th of June, detailed individual data have been recorded through the Clinical survey for 17,025 patients by 97 general hospitals which reported more than 99% of the cases and by 19 other – mainly psychiatric – hospitals for less than 1% of the cases. Both admission and discharge questionnaires were filled in for 12,891 patients (76%). Admission information had not been reported for 1513 (9%) of the patients and discharge information for 2621 (15%) of the patients. Patients not yet reported as discharged include the patients still hospitalized and those discharged but not reported yet on the 28th of June. Compared to the patients with both admission and discharge information, those without discharge information were slightly older (mean 67.2 vs. 68.5 years, respectively), but there were no significant differences regarding sex and most comorbidities (see Additional file 3). Those with no admission information had no significant differences regarding age and sex, but experienced slightly more stays in ICU (13.5% vs. 15.2%, respectively) and a higher proportion of deaths (20.8% vs. 25.5%, respectively). Missingness for key demographic variables (sex: 0.8% and age: 1.2%) in the Clinical survey is very low.

To assess coverage of the Clinical survey, we compared the number of admitted cases recorded with the results of the exhaustive SC survey. In view of comparing similar patients, only laboratory-confirmed patients reported by general hospitals were included, patients transferred from another hospital or readmitted were excluded. The estimated coverage on the period 15th of March - 27th of June was 71% (Table 1). During week 12 and 13, the number of patients reported in the Clinical survey exceeded the number of new lab-confirmed admissions as reported through the SC survey. Since week 18, the coverage of the Clinical survey falls below 50%, most probably due to a delay in reporting.

**Table 1:** Number of patients with laboratory-confirmed COVID-19 infection admitted to general hospitals, per epidemiological week, as reported in the Clinical survey and in Surge Capacity survey up to the 27th of June 2020, Belgium

Epidemiological week of diagnosis	Patients included in the Clinical survey^a^; N (%)	Total patients newly admitted in Belgian hospitals (SC survey)^b^
W 8 (16–22/02/2020)	2	NA
W 9 (23–29/02/2020)	7	NA
W 10 (30/02–07/03/2020)	23	NA
W 11 (8–14/03/2020)	219	NA
W 12 (15–21/03/2020)	1308 (102%)	1277
W 13 (22–28/03/2020)	3290 (104%)	3171
W 14 (29/03–04/04/2020)	2986 (80%)	3731
W 15 (05–11/04/2020)	2099 (72%)	2897
W 16 (12–18/04/2020)	1238 (63%)	1960
W 17 (19–25/04/2020)	805 (53%)	1507
W 18 (26/04–02/05/2020)	487 (45%)	1083
W 19 (03–09/05/2020)	351 (43%)	818
W 20 (10–16/05/2020)	238 (38%)	627
W 21 (17–23/05/2020)	154 (30%)	506
W 22 (24–30/05/2020)	106 (30%)	354
W 23 (31/05–06/06/2020)	55 (21%)	265
W 24 (07–13/06/2020)	60 (26%)	229
W 25 (14–20/06/2020)	24 (14%)	169
W 26 (21–27/06/2020)	14 (10%)	144

^a^ Number of patients recorded in the Clinical survey, admitted to general hospitals with a laboratory-confirmed COVID-19 (excluding patients diagnosed by chest computed tomography scan only) who were not referred from another hospital nor readmitted after a first COVID-19 related hospitalization

^b^ Number of patients admitted to the hospital with a COVID-19 laboratory-confirmation available at the moment of reporting (including both COVID-19-related admissions and hospital admissions for other reasons diagnosed with COVID-19 through screening) and who were not referred from another hospital. All general hospitals in Belgium are included in the Surge Capacity survey. Starting from the 15th of March, > 99% of hospitals were reporting daily

Comparison of the mortality figures from the Clinical survey with SC survey, restricted to those laboratory-confirmed to compare similar populations, showed no significant difference in sex and age distribution, but a slightly higher proportion of death reported in the Clinical survey (21.0 and 19.8%, respectively; *p* = 0.015) (see Additional file 4).

## Discussion

In this epidemic with a novel virus, it was of great importance to rapidly collect as much accurate information as possible in order to characterize the public health threat and support the health authorities in its management. This national double-level hospital-based surveillance was implemented very rapidly after the first COVID-19 patients were hospitalized and revealed to be crucial. The two approaches are complementary and serve different needs, for different end-users.

First, SC survey data allows a daily follow-up of Belgian hospital capacity. This close follow-up of the daily capacity enables rapid interventions regarding the potential saturation of local hospital networks. The HTSC Committee, together with the federal hygiene inspectors, regulates transportation of patients based on the individual situation within the hospitals. The close follow-up also allows to regulate the restart of elective activities within the hospitals. The occupancy rate of ICU beds is considered as the most important indicator during the different phases of the lockdown de-escalation plan. The ICU bed capacity for COVID-19 patients is regulated by formalizing an ICU bed surge capacity. Further, the data on ICU, IV and ECMO are used for logistic purposes, e.g. supply management by the Federal Agency for Medicines and Health Products. For the purpose of hospital capacity monitoring, it is very important that patients suspected of having COVID-19, in addition to those confirmed, are also registered, since they also occupy beds in the isolation units. Despite the fact that the SC survey was originally intended for operational purposes, the collected data allows the monitoring of epidemiological trends in hospitals as well. The surveillance of COVID-19 mortality takes advantage of the mandatory nature of the SC survey for the reporting of hospital deaths due to COVID-19. The daily reporting by all Belgian hospitals, ensures exhaustiveness of in-hospital deaths due to COVID-19.

The individual patient data obtained through the Clinical survey allows to study the different in-hospital care trajectories and lengths of stay, as well as insights into risk groups and patient outcomes. Specific research questions could be addressed such as defining priority groups for vaccination, assessing the effectiveness of treatment regimens (e.g. hydroxychloroquine), characterizing certain patient groups (e.g. pediatrics), studying epidemiological and clinical differences among hospitalized patients associated with health inequalities (approximated by ethnicity and postal code), etc. Furthermore, supplementary ad hoc analyses are performed to respond to questions raised by health authorities such as proportions, characteristics and outcomes of patients in ICU and ECMO, proportion of non-Belgian residents among hospitalized patients, proportions and outcomes of hospitalized healthcare workers and residents in nursing homes.

Between March 14th (date of the first epidemiological bulletin) and June 30th, 94 daily epidemiological bulletins were published in which daily trends of key indicators (e.g. number of hospitalizations, number of admissions, and number of discharges) obtained through the SC survey were presented. Moreover, during the same period, additional information on hospitalized patients’ characteristics and risk factors for hospitalization and death obtained through the Clinical survey were presented in 12 weekly bulletins. These two different bulletins aimed to inform both authorities and the general public about the evolution of the epidemic and are made available on the dedicated COVID-19 Sciensano web page (https://covid-19.sciensano.be/fr/covid-19-situation-epidemiologique). During this period, there were more than 600,000 downloads of the daily bulletin. Since the SC survey underwent several changes over time, the presentation of data in the daily bulletin was adapted accordingly, which was sometimes a challenge, as these changes were not always easily understood by the general public. In order to tackle this, a FAQ on the surveillance systems and the way of reporting its data is also made available. An additional thematic report on the highlights of this double surveillance in hospitals was published recently [16]. Supplementary aggregated information at national and provincial level is available on the EpiStat website [17]. Further, the data from both surveys are used for external scientific efforts such as modelling different short and long term scenarios in the context of the exit strategy.

Next to serving needs at the national, regional, or provincial level, the collected data over time proved to be useful at hospital level as well. Individual hospitals consult the statistics to better assess the epidemiological situation in their province and use them for their own planning. Findings related to clinical and care aspects are provided to the hospital practitioners to support them in the management of the COVID-19 patients. Exports of individual hospital data from the SC survey is often requested for internal evaluations. Specific reports for hospitals on the Clinical survey results were distributed every 2 to 3 weeks from the beginning of April, with the aim of informing caregivers about the clinical characteristics and hospital evolution of COVID-19 patients an supporting them in their management. From the 29th of April, the data recorded by each hospital is transferred back to them after data cleaning and management, via the eHealthbox, to be used for hospitals’ own analyses and statistics. This return of information to the data providers partly compensates for the heavy registration burden.

These data are also intended to be used for scientific publications by multidisciplinary teams in national and international projects. A Belgian collaborative group on hospital COVID-19 surveillance, including representatives of the most actively reporting hospitals in the Clinical survey and scientists from Sciensano, was created with the aim to establish a forum to discuss about scientific aspects of the surveillance and to exchange views on topics of interest that could be further analyzed.

This surveillance has both strengths and limitations. A major strength is the rapidity of implementation of both surveys. Another major advantage is the exhaustiveness of the SC survey thanks to the mandatory participation of the general hospitals as supported by a legal framework. This allows a complete and timely overview on the situation in the hospitals throughout the country. Although not mandatory, the Clinical survey is strongly recommended and this resulted in a high participation rate thanks to the engagement of the hospitals. Nevertheless, we observed missing admission or discharge report forms for a sizeable proportion of patients in the Clinical survey, but the absence of difference for most baseline characteristics for subgroups with missing data, comforted us in the reliability and generalizability of the findings. The comparison of the mortality data reported in the Clinical and the SC surveys confirmed representativeness of the Clinical survey for baseline demographic characteristics of the deceased patients. But a very slightly higher mortality was estimated in the Clinical survey, particularly among those with only discharge information, maybe due to a tendency to report more those deceased given the well-established practice of mandatory reporting in hospitals.

A specific limitation of the SC survey is that there is no clear view on the number of CT-scan-confirmed patients, as they are registered together with the possible cases. Only at a later stage, a distinction was made concerning the new admissions. Another limitation of the SC survey is that the daily number of new lab-confirmed COVID-19 hospitalizations are underestimated. The exceeding coverage of the Clinic survey with regard to the SC survey in week 12 and 13 indicates misclassification of lab-confirmed cases in the SC survey at the start of the epidemic in Belgium. Indeed, test results are not always available at the moment of reporting. COVID-19 patients newly admitted to the hospitals but with pending test results are reported in the category ‘CT-confirmed/suspected’ of the survey. When the test result turns out positive, these patients are reclassified and included in the prevalence number of lab-confirmed COVID-19 patients in the hospital, leading to a discrepancy between the incidence and prevalence figures.

A limitation of both surveillances is the continuous changing of variables which impacted the profile of reported patients over time. Important information was not captured from the start, such as the number of COVID-19 patients coming from nursing homes, distinguishing between real COVID-19 cases and COVID-19 positive patients diagnosed through screening, ethnicity, etc. However, the flexibility by which the data collection was adapted to incorporate such variables and promptly react to evolving aspects of the epidemic, modifications of the case definition, and changes in testing strategies is a clear strength of both surveys. Similarly, additional variables were added to enable the investigation of specific research questions, such as the addition of questions more specific for ICU and pediatric patients. We are conscious of the fact that the reporting is a supplementary burden for hospitals already under pressure during the COVID-19 crisis. Particular attention was devoted to ensure a trade-off between information gain (adding variables to the survey) and assuring compliance. The changes in case definition were particularly challenging for data providers to accurately classify their patients. With a similar objective of facilitating access to reporting, an access code was not set up at the initiation of the surveys but later on when it appeared necessary to ensure the integrity of the encoded responses and when the reporting habit was already well established. Clear communication on the changes and support was available throughout the process and widely used to help data providers report in a correct way. All analyses and interpretations of results have taken into account the timing of survey modifications.

## Conclusion

Through the establishment of this double COVID-19 surveillance, a solid system of hospital data collection was put in place to serve public health needs. There is still a lot to be learned on COVID-19 and the information collected within this surveillance will contribute further to understanding various care-related and epidemiological aspects of the disease. The 2 surveys are flexible and will allow to adapt to the future evolution of the epidemic. Furthermore the registration of the patient’s national registry number, under a pseudonimized format, in the Clinical survey will allow future linkages of this database, in respect of patients’ privacy, with other national data sources such as Belgium’s civil registry or the Belgian Intermutualistic Agency (IMA-AIM) database which includes all administrative records of healthcare related expenses. These database merges, as well as possible studies by direct contact of registered patients, will help to estimate the psychological, physical or social consequences of severe COVID-19 infections in the medium and long term.

## Supplementary Information

**Additional file 1.** Timeline of changes applied to the data collection in the Surge Capacity survey in Belgium, February – June 2020. Scheme of the changes applied to the data collection in the Surge Capacity survey with their respective dates of implementation.

**Additional file 2.** Variables list, description and data inclusion/removal of the Clinical survey admission and discharge forms. List of the variables in the Clinical survey with their date of addition/removal.

**Additional file 3.** Comparison baseline characteristics of patients in the Clinical survey based on the availability of admissions and/or discharge form. Baseline characteristics of patients in the Clinical survey with both discharge and admission forms vs patients without discharge form, and patients without admission form.

**Additional file 4.** Comparison of mortality figures from the Clinical survey with the Surge Capacity survey. Comparison of the proportion of lab-confirmed deaths reported and the age and sex of lab-confirmed deceased persons between the Clinical survey and the Surge Capacity survey.

## Data Availability

The data obtained through both surveillance systems are available from the corresponding author on reasonable request according to Sciensano scientific policy and after approval by the Belgian data protection authority.

## Abbreviations

ACE: Angiotensin-Converting Enzyme
ARBs: Angiotensin II Receptor Blockers
COVID-19: Coronavirus disease 2019
CT: Computed tomography
ECDC: European Centre for Disease Prevention and Control
ECMO: Extracorporeal membrane oxygenation
FAQ: Frequently Asked Questions
GDPR: General Data Protection Regulation
HTSC: Hospital & Transport Surge Capacity
ICU: Intensive Care Unit
IMA-AIM: Intermutualistic Agency
IV: Invasive Ventilation
LTCF: Long-Term Care Facilities
RMG: Risk Management Group
RT-PCR: Reverse Transcriptase–Polymerase Chain Reaction
SARS-CoV-2: Severe acute respiratory syndrome coronavirus 2
SC: Surge Capacity
WHO: World Health Organization
