# Correction to: Rapid establishment of a national surveillance of COVID-19 hospitalizations in Belgium

**DOI:** 10.1186/s13690-021-00661-w

**Published:** 2021-08-18

**Authors:** Nina Van Goethem, Aline Vilain, Chloé Wyndham-Thomas, Jessika Deblonde, Nathalie Bossuyt, Tinne Lernout, Javiera Rebolledo Gonzalez, Sophie Quoilin, Vincent Melis, Dominique Van Beckhoven

**Affiliations:** 1 Scientific Directorate of Epidemiology and Public Health, Sciensano, J. Wytsmanstraat 14, 1050 Brussels, Belgium; 2 Department of Epidemiology and Biostatistics, Institut de recherche expérimentale et clinique, Faculty of Public Health, Université catholique de Louvain, Clos Chapelle-aux-champs 30, 1200 Woluwe-Saint-Lambert, Belgium; 3 Directorate Healthcare, Federal Public Service (FPS) Health, Food Chain Safety and Environment, Brussels, Belgium

**Correction to: Arch Public Health 78, 121 (2020)**

**https://doi.org/10.1186/s13690-020-00505-z**

Following publication of the original article [1], the author reported an error in the affiliations:

The original first affiliation was

1. Public health and genome, Scientific Directorate of Epidemiology and public health, Sciensano, J. Wytsmanstraat 14, 1050 Brussels, Belgium

The correct affiliation has been provided in this Correction.

1. Scientific Directorate of Epidemiology and public health, Sciensano, J. Wytsmanstraat 14, 1050 Brussels, Belgium

The original article [1] has been updated.
