# Supplementary material for: Rapid establishment of a national surveillance of COVID-19 hospitalizations in Belgium
Source: Arch Public Health. 2020 Nov 18;78:121. doi: 10.1186/s13690-020-00505-z (PMC7673251; doi:10.1186/s13690-020-00505-z)
Supplement: Supplementary file 1 — Additional file 1. Timeline of changes applied to the data collection in the Surge Capacity survey in Belgium, February – June 2020. Scheme of the changes applied to the data collection in the Surge Capacity survey with their respective dates of implementation. [file 13690_2020_505_MOESM1_ESM.pdf]

Changes applied to data collection in the Surge Capacity survey

		10th of March		24th of March	11th of April	28th of April	26th of May	20th of June
Prevalence figures	Laboratory-confirmed; CT-confirmed/suspected	No hospitalized patients in hospital						
		No hospitalized patients in ICU						
		No hospitalized patients under IV						
		No hospitalized patients under ECMO						
Incidence figures	Laboratory-confirmed	No hospital admissions / last 24 hrs	Not referred from other hospital			Not referred from NH/LTCF/other hospital		
						Referred from NH/LTCF		
			Referred from other hospital			Referred from other hospital		
						Admitted for other pathology		
		No hospital discharges / last 24 hrs	Not referred from other hospital					
			Referred from other hospital					
		No deaths / last 24 hrs		+ DoD, age, gender	+ DoB, postal code, method of diagnosis			+ collectivity
	CT-confirmed / suspected	No hospital admissions / last 24 hrs	Not referred from other hospital			Not referred from NH/LTCF/other hospital	CT-confirmed cases	
						Referred from NH/LTCF	Possible cases	
			Referred from other hospital			Referred from other hospital	Referred from NH/LTCF	
						Admitted for other pathology	Referred from other hospital	
		No hospital discharges / last 24 hrs	Not referred from other hospital					
			Referred from other hospital					
		No deaths / last 24 hrs		+ DoD, age, gender	+ DoB, postal code, method of diagnosis			+ collectivity

IV=invasive ventilation; DoD=date of death; DoB=date of birth; NH=nursing home; LTCF=long-term care facility

The SC survey has been operational since the 10th of March 2020. The following variables are collected as a prevalence number: the number of hospitalized COVID-19 patients in all units of the hospital (including ICU), in the ICU, under invasive ventilation (IV), and under Extra-Corporeal Membrane Oxygenation (ECMO). The following variables are collected as an incidence number: the number of new COVID-19 hospital admissions in the last 24h, the number of COVID-19 discharges in the last 24h, and the number of COVID-19 deaths in the last 24h. All those variables are collected separately for the lab-confirmed cases ('lab-confirmed' category) on the one hand, and CT-confirmed and possible cases ('CT-confirmed/suspected' category) on the other hand.

For incidence numbers of admissions and discharges, a distinction is made between patients referred from/to another hospitals and those that are not. Successive stratifications were added to the number of hospital admissions. To have a more complete view on the origin of the patients, patients coming from a nursing home or another long-term care facility (LTCF) are recorded separately from the 28th of April onwards. In view of the Risk Management Group (RMG) advice of the 22nd of April broadening the indication criteria for COVID-19 molecular testing (all patients admitted to a hospital can be tested, regardless of the reason for hospital admission), the gradual restart of normal hospital activities, and the implementation of COVID-19 screening policies in numerous hospitals, the number of hospital admissions was stratified according to indication for testing on the 28th of April. This has allowed to differentiate COVID-19-related admissions from hospital admissions for other reasons diagnosed with COVID-19 through screening.

On the 26th of May, in order to have a view on the number of CT-confirmed cases, the number of hospital admissions in the 'CT-confirmed/suspected' category of the survey was split-up between CT-confirmed cases and possible cases.

Starting from the 24th of March, individual data collection was added for each confirmed and suspected COVID-19 patient who died as a result of a COVID-19 infection, including the date of death, age, and gender of the patient. On the 11th of April, the exact date of birth and the postal code of the residence of the patient was added to facilitate the removal of double entries and efficient linking with other mortality databases in the future. In addition, the precise method of COVID-19 diagnosis was added for each of the deaths to be able to distinguish, next to the lab-confirmed deaths, the CT-confirmed deaths from the suspected ones. On the 20th of June, a variable was added to indicate whether or not the deceased patient originated from a collectivity, to have a view on the number of residents from nursing homes or other institutions dying at the hospital.
