# Supplementary material for: Rapid establishment of a national surveillance of COVID-19 hospitalizations in Belgium
Source: Arch Public Health. 2020 Nov 18;78:121. doi: 10.1186/s13690-020-00505-z (PMC7673251; doi:10.1186/s13690-020-00505-z)
Supplement: Supplementary file 2 — Additional file 2. Variables list, description and data inclusion/removal of the Clinical survey admission and discharge forms. List of the variables in the Clinical survey with their date of addition/removal. [file 13690_2020_505_MOESM2_ESM.docx]

**Annex 2: Supplementary table**: **Variables list, description and data inclusion/removal of the Clinical survey admission and discharge forms.**

*In the online questionnaire some question appear only conditionally of other questions’ answer.*

| Variable name | Description | Value set or terminology | Date inclusion or removal^[[1]](#footnote-1)^ |
| --- | --- | --- | --- |
| Admission questionnaire | | | |
| dt_reporting_admis | Declaration date | Date |  |
| Hospital_name_admis | Hospital name | Dropdown list |  |
| Hosp_patient_ID | Hospital record ID of the patient | Text |  |
| National_nr | National registry number of the patient - pseudonimized | Text | From Sept. 14 |
| dob | Birth date of patient | Date |  |
| gender | Gender or patient | Male/Female/Other/ Unknown |  |
| Post_code | Post code of patient (in Belgium) | Numeric |  |
| Country_resid | Country of residence | Belg./Unknown/Other | From June 8 |
| country_resid_other | Specification of country of residence, if 'other' | Text | From June 8 |
| Readmission | Previous hospitalization for COVID-19 and readmission | Yes/No | From April 22 |
| Prev_admis_same_hosp | If readmitted for covid, from same hospital? | Yes/No | From April 22, up to Sept. 14 |
| Prev_admis_hosp_patient_id | if same hospit, hospital patient id from previous hospital stay | Text | From April 22, up to Sept. 14 |
| Prev_admis_hospital_name | if readmitted after discharge from other hospital, hospital name | Dropdown list | From April 22, up to Sept. 14 |
| Prev_admis_dt_discharge | if readmitted after discharge from other hospital, date discharge | Date | From April 22, up to Sept. 14 |
| Expo_HCW | Patient is a health care worker | Yes/No/ Unknown |  |
| Expo_retirement_home | Patient is a nursing home resident | Yes/No/Unknown |  |
| Expo_collective_housing | Patient is a resident in a collective housing | No / Yes, revalidation centre / Yes, psychiatric centre / Yes, homeless housing / Yes, other / Unknown | From June 8, up to Sept. 14 |
| IndexCase | Index case in the HH (< 18)^[[2]](#footnote-2)^ | Patient / Yes, adult / Yes, child / Unknown | From June 8 |
| N_household | Number of persons living in the same household (<18) | Numeric | From June 8 |
| Ethnic | Ethnic group of the patient | European /Hispanic / Asian / Subsaharan African / North African / Mixed / Other / Unknown | From June 8 |
| Test_for_covid | Test for symptoms suggestive of COVID | Yes/No | From June 8 |
| Test_systematic | Systematic testing of an hospitalized patient | Yes/No | From June 8 |
| Test_other | Test for other reason | Yes/No | From June 8 |
| Test_unknown | Unknown reason for testing | Yes/No | From June 8 |
| Expo_travel | Exposition - Travel to risk region | Yes/No | Up to June 8 |
| Expo_Contact_confirmed | Exposition - Contact with confirmed COVID-19 patient | Yes/No | Up to Sept. 14 |
| Expo_contact_probable | Exposition - Contact with probable COVID-19 patient | Yes/No | Up to Sept. 14 |
| Expo_nosocomial | Exposition - Suspicion of nosocomial infection^[[3]](#footnote-3)^ | Yes/No | Up to Sept. 14 |
| Expo_no_identified | Exposition - Not identified^[[4]](#footnote-4)^ | Yes/No | Up to Sept. 14 |
| Expo_unknown | Exposition – Unknown ^[[5]](#footnote-5)^ | Yes/No | Up to Sept. 14 |
| Expo_other | Exposition - Other | Text field | Up to Sept. 14 |
| Dt_onset | Onset date of the symptoms | Date |  |
| Dt_admission | Date of hospital admission | Date |  |
| Reason_clinical_status | Reason hospitalisation - Clinical status related to COVID-19 | Yes/No | Up to Sept. 14 |
| Reason_at_risk | Reason hospitalisation - Person at risk for COVID-19 | Yes/No | Up to Sept. 14 |
| Reason_entourage | Reason hospitalisation - Entourage at risk for COVID-19 | Yes/No | Up to Sept. 14 |
| Reason_imposs_at_home | Reason hospitalisation - Isolation at home impossible | Yes/No | Up to Sept. 14 |
| Reason_noncov_hospit | Reason hospitalisation - Hospitalisation for another reason no related to COVID | Yes/No | From June 8, up to Sept. 14 |
| Reason_transfer | Reason hospitalisation - Transfer from other hospital | Yes/No |  |
| Transfer_from | Transfer from which hospital | Dropdown list |  |
| transfer_from_other | Specification of hospital name, if other | Text |  |
| Reason_other | Reason hospitalision - Other | Yes/No | Up to Sept. 14 |
| Symptom_fever | Symptom at admission - Fever | Yes/No |  |
| Symptom_viral | Symptom at admission – Viral syndrome: including previously collected weakness, headache, muscle & joint pain | Yes/No | From Sept. 14 |
| Symptom_weakness | Symptom at admission - General weakness | Yes/No | Up to Sept. 14 |
| Symptom_upper_respi | Symptom at admission of upper respiratory tract infection: including previously collected throat pain, runny nose | Yes/No | From Sept. 14 |
| Symptom_low_respi | Symptom at admission of low respiratory tract infection: including previously collected cough, breathlessness, thoracic pain | Yes/No | From Sept. 14 |
| Symptom_GI | Symptom at admission – gastro-intestinal: including previously collected diarrhea, nausea / vomiting, abdominal pain |  | From Sept. 14 |
| Symptom_cough | Symptom at admission - Cough | Yes/No | Up to Sept. 14 |
| Symptom_throat_pain | Symptom at admission - Throat pain | Yes/No | Up to Sept. 14 |
| Symptom_runny_nose | Symptom at admission - Runny nose | Yes/No | Up to Sept. 14 |
| Symptom_anosmia | Symptom at admission - anosmia- agueusia | Yes/No | From march 23 |
| Symptom_breathless | Symptom at admission - Breathlessness | Yes/No | Up to Sept. 14 |
| Symptom_diarrhoea | Symptom at admission - Diarrhoea | Yes/No | Up to Sept. 14 |
| Symptom_nausea_vomit | Symptom at admission - Nausea | Yes/No | Up to Sept. 14 |
| Symptom_loss_apetite | Symptom at admission: reduced feeding (< 18) | Yes/No | From June 8 |
| Symptom_headache | Symptom at admission - Headache | Yes/No | Up to Sept. 14 |
| Symptom_mental | Symptom at admission - Irritability | Yes/No | Up to Sept. 14 |
| Symptom_pain | Symptom at admission - Pain | Yes/No | Up to Sept. 14 |
| Symptom_rash | Symptom at admission - Skin rash (< 18) | Yes/No | From June 8 |
| Symtom_erythema | Symptom at admission - Erythema multiforme (< 18) | Yes/No | From June 8 |
| Symptom_urtic | Symptom at admission - Urticaria (< 18) | Yes/No | From June 8 |
| Symptom_petechia | Symptom at admission - Petechia (< 18) | Yes/No | From June 8 |
| Pain_muscle | Pain location - Muscles | Yes/No | Up to Sept. 14 |
| Pain_abdomen | Pain location - Abdomen | Yes/No | Up to Sept. 14 |
| Pain_thorax | Pain location - Thorax | Yes/No | Up to Sept. 14 |
| Pain_joint | Pain location - Joint | Yes/No | Up to Sept. 14 |
| Pain_other | Pain location - Other | Yes/No | Up to Sept. 14 |
| Symptom_none | Symptom at admission - None | Yes/No |  |
| Symptom_other | Symptom at admission - Other | Text field |  |
| Clin_sign_pharyngitis | Clinical sign - Pharyngeal exsudate | Yes/No | From Sept. 14 for < 18 only |
| Clin_sign_conjonctivitis | Clinical sign - Conjonctival injection | Yes/No | From Sept. 14 for < 18 only |
| Clin_sign_convulsions | Clinical sign - Convulsions | Yes/No | From Sept. 14 for < 18 only |
| Clin_sign_coma | Clinical sign - Coma | Yes/No | From Sept. 14 for < 18 only |
| Clin_sign_dyspnea | Clinical sign - Dyspnea | Yes/No | From Sept. 14 for < 18 only |
| Clin_sign_lung_auscult_abnorm | Clinical signs - Abnormal pulmonary auscultation | Yes/No | From Sept. 14 for < 18 only |
| Clin_sign_lung_Xray_abnorm | Clinical signs - Abnormal pulmonary imaging compatible with viral pneumonia | Yes/No | From Sept. 14 for < 18 only |
| Clin_sign_none | Clinical signs - None | Yes/No | From Sept. 14 for < 18 only |
| Clin_sign_Other | Clinical signs - Other | Text field | From Sept. 14 for < 18 only |
| Temperature | Temperature | Numeric | From Sept. 14 for < 18 only |
| Freq_cardiac | Cardiac frequency (< 18) | Count/min - numeric | From June 8 |
| Freq_respi | Respiratory frequency (< 18) | Count/min - numeric | From June 8 |
| BP_syst | Systolic Blood pressure (< 18) | Numeric (mmHg) | From June 8 |
| BP_diast | Diastolic Blood pressure (< 18) | Numeric (mmHg) | From June 8 |
| Value_SpO2_admission | Value of SpO2 at hospital admission (< 18) | Numeric (%) | From June 8 |
| Comorbid_CVD | Comorbidities - Cardiovasular disease |  |  |
| Comorbid_HBP | Comorbidities - Arterial hypertension | Yes/No |  |
| Comorbid_diabetes | Comorbidities - Diabetes | Yes/No |  |
| Comorbid_renal_chronic | Comorbidities - Chronic renal disease | Yes/No |  |
| Comorbid_liver_chronic | Comorbidities - Chronic liver disease | Yes/No |  |
| Comorbid_neuro_chronic | Comorbidities - Chronic neurologic or neuromuscular disease (with the exception of cognitive troubles)^[[6]](#footnote-6)^ | Yes/No |  |
| Comorbid_cognitive | Comorbidities - Cognitive troubles | Yes/No | From March 23 |
| Comorbid_immunodep | Comorbidities - Immunodepresion, including HIV | Yes/No |  |
| Comorbid_lung_chronic | Comorbidities - Chronic lung disease | Yes/No |  |
| Comorbid_solid_cancer | Comorbidities - Solid cancer | Yes/No |  |
| Comorbid_hemato_cancer | Comorbidities - Hematological cancer | Yes/No |  |
| Comorbid_transplant | Comorbidities - Solid organ transplantation | Yes/No | From June 8 |
| Comorbid_pregnancy | Comorbidities - Pregnancy | Yes/No |  |
| Comorbid_postpartum | Comorbidities - Post partum (< 6 weeks) | Yes/No |  |
| Comorbid_premature | Comorbidities - Prematurity | Yes/No | From Sept. 14 for < 18 only |
| Comorbid_obesity | Comorbidities - Obesity | Yes/No | From April 3 |
| Comorbid_none | Comorbidities - None | Yes/No |  |
| Comorbid_other | Comorbidities - Other | Yes/No | From April 3 |
| Cardiac_failure | If CVD, cardiac failure? (< 18) | Yes/No | From June 8 |
| Lung_details | If comorbid_lung_chronic = "Yes", details (< 18) | Cystic fibrosis / Severe asthma / Bronchiectasis / Ciliary diskynesia / tuberculosis / dysplasia / Other / Unknown | From June 8 |
| Immuno_details | If immunodepression = "Yes", details (< 18) | HIV / primary immunodeficiency / immunosuppressive treatment / Other / Unknown | From June 8 |
| Transplant_organ | If comorbid_transplant = "Yes", which organ? (< 18) | Kidney / Liver / Lung / Heart / Other | From June 8 |
| Dt_transplant | If comorbid_transplant = "Yes", date of transplantation? (<18) | date | From June 8 |
| Solid_cancer_active | If comorbid_solid_cancer = "Yes", active in last 5 years? | Yes/No/Unknown | From June 8 |
| S_cancer_treat6M | If comorbid_solid_cancer = "Yes", treatment in the last 6 months? | Yes/No/Unknown | From June 8, up to Sept. 14 |
| Cancer_bladder | if comorbid_solid_cancer = "Yes", cancer of bladder | Yes/No | From June 8, up to Sept. 14 |
| Cancer_breast | if comorbid_solid_cancer = "Yes", cancer of breast | Yes/No | From June 8, up to Sept. 14 |
| Cancer_colon | if comorbid_solid_cancer = "Yes", cancer of colon/rectum | Yes/No | From June 8, up to Sept. 14 |
| Cancer_endometrium | if comorbid_solid_cancer = "Yes", cancer of endometrium | Yes/No | From June 8, up to Sept. 14 |
| Cancer_headneck | if comorbid_solid_cancer = "Yes", cancer of head & neck | Yes/No | From June 8, up to Sept. 14 |
| Cancer_kidney | if comorbid_solid_cancer = "Yes", cancer of kidney | Yes/No | From June 8, up to Sept. 14 |
| Cancer_liver | if comorbid_solid_cancer = "Yes", cancer of liver | Yes/No | From June 8, up to Sept. 14 |
| Cancer_lung | if comorbid_solid_cancer = "Yes", cancer of lung | Yes/No | From June 8, up to Sept. 14 |
| Cancer_melanoma | if comorbid_solid_cancer = "Yes", melanoma | Yes/No | From June 8, up to Sept. 14 |
| Cancer_pancreas | if comorbid_solid_cancer = "Yes", cancer of pancreas | Yes/No | From June 8, up to Sept. 14 |
| Cancer_prostate | if comorbid_solid_cancer = "Yes", cancer of prostate | Yes/No | From June 8, up to Sept. 14 |
| Cancer_thyroid | if comorbid_solid_cancer = "Yes", cancer of thyroid | Yes/No | From June 8, up to Sept. 14 |
| Cancer_ovarian | if comorbid_solid_cancer = "Yes", cancer of ovary | Yes/No | From June 8, up to Sept. 14 |
| Cancer_other | if comorbid_solid_cancer = "Yes", other cancer | Text field | From June 8, up to Sept. 14 |
| Cancer_unknown | if comorbid_solid_cancer = "Yes", unknown location of cancer | Yes/No | From June 8, up to Sept. 14 |
| Hemato_cancer_active | If comorbid_hemato_cancer = "Yes", active in last 5 years? | Yes/No/Unknown | From June 8 |
| H_cancer_treat6M | If comorbid_hemato_cancer = "Yes", treatment in the last 6 months? | Yes/No/Unknown | From June 8, up to Sept. 14 |
| H_Cancer_hodgkin | if comorbid_solid_cancer = "Yes", Hodgkin lymphoma | Yes/No | From June 8, up to Sept. 14 |
| H_Cancer_leukemia | if comorbid_solid_cancer = "Yes", leukemia | Yes/No | From June 8, up to Sept. 14 |
| H_Cancer_myeloma | if comorbid_solid_cancer = "Yes", multiple myeloma | Yes/No | From June 8, up to Sept. 14 |
| H_Cancer_non_hodgkin | if comorbid_solid_cancer = "Yes", non hodgkin lymphoma | Yes/No | From June 8, up to Sept. 14 |
| H_Cancer_other | if comorbid_solid_cancer = "Yes", other | Text field | From June 8, up to Sept. 14 |
| H_Cancer_unknown | if comorbid_solid_cancer = "Yes", unknown type of cancer | Yes/No | From June 8, up to Sept. 14 |
| Hemato_stemcell | if comorbid_hemato_cancer = "Yes", patient received stem cell transplantation? (< 18) | Yes/No | From June 8 |
| Dt_stemcell | if hemato_stemcell = "Yes", date of transplantation (< 18) | date | From June 8 |
| Prema_preg_weeks | if comorbid_premature = "Yes", weeks of pregnancy at birth (< 18) | Numeric (weeks) | From June 8 |
| Prema_birth_weight | if comorbid_premature = "Yes", weight at birth | Numeric (gr) | From June 8 |
| Pregnancy_trimester | Pregnancy trimester (< 18) | 1st/2nd/3rd/Unknown |  |
| Current_smoker | Current smoker? | Yes/No/Unknown |  |
| Influenza_vaccine | Has the patient been vaccinated against influenza (season 2019-2020)? | Yes/No/Unknown | Up to Sept. 14 |
| Treat_IEC_Sartan | Is the patient under IEC and/or SARTAN treatment? | Yes, an IEC / Yes, a SARTAN / Yes, an IEC and a SARTAN / No / Unknown | Up to Sept. 14 |
| Treat_NSAI | Is the patient under usual treatment NSAI? (< 18) | Yes/No/Unknown | From June 8 |
| Treat_corticoid_PO | Is the patient under usual treatment with oral corticoids? (< 18) | Yes/No/Unknown | From June 8 |
| Cortic_dose | if Treat_corticoid_PO="Yes" then which dose. (< 18) | Numeric (/kg/day) | From June 8 |
| Treat_immunodep | Is the patient under usual treatment with immunodepressor? (< 18) | Yes/No/Unknown | From June 8 |
| Lab_name | Laboratory name | Text | Up to April 3 |
| Dt_sample | Date of sample for COVID-19 diagnosis | Date | Up to April 3 |
| Type_sample | Type of sample | Nasopharyngeal swab / nasopharyngeal aspirate / BAL / Other | Up to April 3 |
| Type_sample_other | Specification of type of sample, if other |  | Up to April 3 |
| Dt_lab_confirmation | Date of lab confirmation | Date | From April 3 |
| Diag_method_PCR | Diagnostic method - Positive result by PCR? | Yes/No | From April 3 |
| Diag_method_Scan | Diagnostic method - Typical thoracic scan | Yes/No | From April 3 |
| Diag_method_Ag_rapid | Diagnostic method - Positive rapid antigen test | Yes/No | From April 3 |
| Diag_method_Unk | Diagnostic method - Unknown | Yes/No | From April 3 |
| dt_diagnosis | Date du diagnostic ^[[7]](#footnote-7)^ | Date |  |
| Name_admis | Name of the person reporting the admission^[[8]](#footnote-8)^ | Text |  |
| Phone_admis | Phone number of the person reporting the admission^8^ | Text |  |
| Email_admis | Email address of the person reporting the admission^8^ | Text |  |
| Discharge questionnaire | | | |
| Dt_reporting_disch | Declaration date | Date |  |
| Hospital_name_disch | Hospital name | dropdown list |  |
| Pneumonia_Xray | Pneumonia on medical imaging (X-ray or CT-scan)^[[9]](#footnote-9)^ | Yes/No**/**Not done**/**Unknown |  |
| Pneumonia_localisation | if pneumonia_Xray="Yes", Location of pneumonia | Bilateral/Unilateral/Unknown | Up to Sept. 14 |
| X_ray_not_performed | X-ray: not perfomed (< 18) | Yes/No | From June 8 |
| X_ray_normal | X-ray: normal (< 18) | Yes/No | From June 8 |
| X_ray_inters_syndr | X-ray: interstitial syndrome (< 18) | Yes/No | From June 8 |
| X_ray_cardiomegaly | X-ray: cardiomegaly (< 18) | Yes/No | From June 8 |
| X_ray_pleural | X-ray: pleural effusion (< 18) | Yes/No | From June 8 |
| X_ray_consolidation | X-ray: alveolar consolidation (< 18) | Yes/No | From June 8 |
| X_ray_other | X-ray: other image | Yes/No | From June 8 |
| CTscan_not_performed | CT-scan: not perfomed (< 18) | Yes/No | From June 8 |
| CT_scan_normal | CT-scan: normal (< 18) | Yes/No | From June 8 |
| CT_scan_ground_glass | CT-scan: Ground glass (< 18) | Yes/No | From June 8 |
| CT_scan_crazy_paving | CT-scan: crazy paving (< 18) | Yes/No | From June 8 |
| CT_scan_micronodule | CT-scan: micronodules (< 18) | Yes/No | From June 8 |
| CT_scan_pleural | CT-scan: pleural effusion (< 18) | Yes/No | From June 8 |
| CT_scan_consolidation | CT-scan: consolidation (< 18) | Yes/No | From June 8 |
| CT_scan_bronchiectasis | CT-scan:bronchiectasis (< 18) | Yes/No | From June 8 |
| CT_scan_other | CT-scan: other image (< 18) | Yes/No | From June 8 |
| Oxygen | Did the patient receive oxygen during his hospital stay? | Yes/No/Unknown | From June 8 |
| ICU_transfer | Was the patient transferred to ICU | Yes/No/Unknown |  |
| Complic_none | Complication: None | Yes/No | From June 8 |
| ARDS | Complication: ARDS | Yes/No/Unknown | From June 8 |
| Complic_MOF | Complication: MOF | Yes/No | From June 8 |
| Complic_myocarditis | Complication: myocarditis | Yes/No | From June 8, up to Sept. 14 |
| Complic_sepsis | Complication: sepsis | Yes/No | From June 8 |
| Complic_bacterial_super | Complication bacterial superinfection | Yes/No | From June 8, up to Sept. 14 |
| Complic_bacterial_pulmonary | Pulmonary bacterial complication | Yes/No | From Sept. 14 |
| Complic_bacteremia | Complication bacteremia | Yes/No | From Sept. 14 |
| Complic_fungal | Complication: fungal infection | Yes/No | From June 8 |
| Complic_kidney | Complication: kidney failure | Yes/No | From June 8 |
| Complic_delirium | Complication: delirium | Yes/No | From June 8, up to Sept. 14 |
| Complic_conscious | Complication: altered consciousness | Yes/No | From June 8 |
| Complic_shock | Complication: shock | Yes/No | From June 8 |
| Complic_embol | Complication: pulmonary embolism | Yes/No | From June 8 |
| Complic_ACS | Complication: acute coronary syndrome | Yes/No | From June 8 |
| Complic_stroke | Complication: stroke | Yes/No | From June 8 |
| Complic_hep_hypoxia | Complication hepatic hypoxia | Yes/No | From June 8 |
| Complic_other | Complication: other | Text-field | From June 8 |
| Superinfection | Bacterial and/or fungal surinfection | Yes/No/Unknown | Up to June 8 |
| Kawasaki | Kawasaki/SIRS (only < 18) | Yes/No/Unknown | From June 8 |
| Kawasaki_immuno | Treatment for Kawasaki: Immunoglobulines (only < 18) | Yes/No/Unknown | From June 8 |
| Kawasaki_cortic | Treatment for Kawasaki: corticoides (only < 18) | Yes/No/Unknown | From June 8 |
| Kawasaki_AAS | Treatment for Kawasaki: AAS (only < 18) | Yes/No/Unknown | From June 8 |
| Value_PaO2_admission | Value of PaO2 at hospital admission | Numeric |  |
| Value_lympho_admission | Value of Ph at hospital admission | Numeric |  |
| Value_LDH_admission | Value of LDH at hospital admission | Numeric |  |
| Value_CRP_admission | Value of CRP at hospital admission | Numeric |  |
| Value_PCO2_admission | Value of PCO2 at hospital admission | Numeric | Up to June 8 |
| Value_Ph_admission | Value of Ph at hospital admission | Numeric | Up to June 8 |
| Value_lactate_admission | Value of lactate at hospital admission | Numeric | Up to June 8 |
| Creatinine_admission_mg | Value of creatinine in mg/dl at hospital admission | Numeric | Up to June 8 |
| Creatinine_admission_mol | Value of creatinine in µmol/l at hospital admission | Numeric | Up to June 8 |
| Value_PaO2_USI | Value of PaO2 at ICU admission | Numeric | Up to June 8 |
| Value_PCO2_USI | Value of PCO2 at ICU admission | Numeric | Up to June 8 |
| Value_Ph_USI | Value of Ph at ICU admission | Numeric | Up to June 8 |
| Creatinin_admission_USI_mg | Value of creatinine in mg/dl at ICU admission | Numeric | Up to June 8 |
| Creatinin_admission_USI_mol | Value of creatinine in µmol/l at ICU admission | Numeric | Up to June 8 |
| Value_lympho_USI | Value of Lymphocytes at ICU admission | Numeric | Up to June 8 |
| Value_LDH_USI | Value of LDH at ICU admission | Numeric | Up to June 8 |
| Serology_COVID | Serology was performed for the patient? | Yes/No/Unknown | From June 8 |
| Dt_serology | If performed, date of serology | Date | From June 8, up to Sept. 14 |
| Serology_result | Result of serology | Positive/Negative/Undetermined/Unknown | From June 8 |
| Hemoc | Hemoculture (only < 18) | Positive/negative/not performed/unknown | From June 8 |
| Hemoc_germ | if hemoc="positive", which germ was identified (only < 18) | Text field | From June 8 |
| Respi_virus | Coinfection with respiratory virus? | No/Yes/Not tested/Unknown | From June 8 |
| Virus_type | If yes, which virus(es) | Text field | From June 8 |
| Treatment1 | Specific COVID-19 treatment received? | Yes/No/Unknown | Up to June 8 |
| Name_Drug1 | Drug name treatment 1 | Text | Up to June 8 |
| Dt_init_drug1 | Start date treatment 1 | Date | Up to June 8 |
| Dt_stop_drug1 | Stop date treatment 1 | Date | Up to June 8 |
| Drug1_administration | Administration mode drug treatment 1 | Per os/IV/Unknown/Other | Up to June 8 |
| Drug1_admin_other | Specification of other administration mode of drug treatment 1, if 'other' | Text | Up to June 8 |
| Treatment2 | Specific COVID-19 treatment received? | Yes/No/Unknown | Up to June 8 |
| Name_Drug2 | Drug name treatment 2 | Text | Up to June 8 |
| Dt_init_drug2 | Start date treatment 2 | Date | Up to June 8 |
| Dt_stop_drug2 | Stop date treatment 2 | Date | Up to June 8 |
| Drug2_administration | Administration mode drug treatment 2 | Per os/IV/Unknown/Other | Up to June 8 |
| Drug2_admin_other | Specification of other administration mode of drug treatment 2, if 'other' | Text | Up to June 8 |
| Treatment3 | Specific COVID-19 treatment received? | Yes/No/Unknown | Up to June 8 |
| Name_Drug3 | Drug name treatment 3 | Text | Up to June 8 |
| Dt_init_drug3 | Start date treatment 3 | Date | Up to June 8 |
| Dt_stop_drug3 | Stop date treatment 3 | Date | Up to June 8 |
| Drug3_administration | Administration mode drug treatment 3 | Per os/IV/Unknown/Other | Up to June 8 |
| Drug3_admin_other | Specification of other administration mode of drug treatment 3, if 'other' | Text | Up to June 8 |
| HCQ | Hydoxychloroquine received? | Yes/No/Unknown | From June 8 |
| Dt_init_HCQ | Start date HCQ | Date | From June 8, up to Sept. 14 |
| Dt_stop_HCQ | Stop date HCQ | Date | From June 8, up to Sept. 14 |
| Remdesivir | Remdesivir received? | Yes/No/Unknown | From June 8 |
| Dt_init_Remdesivir | Start date remdesivir | Date | From June 8, up to Sept. 14 |
| Dt_stop_remdesivir | Stop date remdesivir | Date | From June 8, up to Sept. 14 |
| Lopi_ritonavir | Lopinavir/Ritonavir received? | Yes/No/Unknown | From June 8, up to Sept. 14 |
| Dt_init_Lopi_rito | Start date Lopinavir/Ritonavir | Date | From June 8, up to Sept. 14 |
| Dt_stop_Lopi_rito | Stop date Lopinavir/Ritonavir | Date | From June 8, up to Sept. 14 |
| Olsetamivir | Olsetamivir received? | Yes/No/Unknown | From June 8, up to Sept. 14 |
| Dt_init_Olsetamivir | Start date Olsetamivir | Date | From June 8, up to Sept. 14 |
| Dt_stop_Olsetamivir | Stop date Olsetamivir | Date | From June 8, up to Sept. 14 |
| Macrolides | Macrolides received? | Yes/No/Unknown | From June 8 |
| Dt_init_Macrolides | Start date Macrolides | Date | From June 8, up to Sept. 14 |
| Dt_stop_Macrolides | Stop date Macrolides | Date | From June 8, up to Sept. 14 |
| Tolicizumab | Tolicizumab received? | Yes/No/Unknown | From June 8, up to Sept. 14 |
| Dt_init_Tolicizumab | Start date Tolicizumab | Date | From June 8, up to Sept. 14 |
| Dt_stop_Tolicizumab | Stop date Tolicizumab | Date | From June 8, up to Sept. 14 |
| Other_treatment | Other medication specific for COVID-19 received? | Yes/No/Unknown | From June 8 |
| Name_other_treat | Name other treatment | Text | From June 8 |
| Dt_init_other_treat | Start date other treatment | Date | From June 8, up to Sept. 14 |
| Dt_stop_other_treat | Stop date other treatment | Date | From June 8, up to Sept. 14 |
| Clinical_trial | Is the patient part of a clinical trial | Yes/No/Unknown | From June 8, up to Sept. 14 |
| AB1 | Antibiotic received during hospitalization? (only < 18) | Yes/No/Unknown | From June 8 |
| AB1_indication | Indication AB 1 (only < 18) | Prophylaxis/Sepsis/Infection KTC /Other | From June 8 |
| AB2 | Second antibiotic received during hospitalization? (only < 18) | Yes/No/Unknown | From June 8 |
| AB2_indication | Indication AB 2 (only < 18) | Prophylaxis/Sepsis/Infection KTC /Other | From June 8 |
| Corticoid_treatment | Systemic corticoid treatment? | Yes/No/Unknown | From June 8 |
| LMWH_prevention | Prevention with low molecular weight heparin | Yes/No/Unknown | From June 8 |
| LMWH_dosis | Dosis of LMWH | Standard/High/Unknown | From June 8 |
| Parent_nutrition | Nutrition parentérale (only < 18) | Yes/No/Unknown | From June 8 |
| Status_discharge | Health status at discharge | Cured/Deceased/Transferred (hospital)/Other^[[10]](#footnote-10)^/Unknown |  |
| Death_covid | If dead, due to covid | Yes/No/Unknown |  |
| Death_nocovid_cause | If dead not due to covid, cause | Text |  |
| Status_discharge_other | Specification of health status at discharge, if 'other' | Text |  |
| Transfer_out_hospit_name | Patient transferred to which hospital? | Dropdown list |  |
| Transfer_out_hospname_other | Name of 'other' hospital the patient was transferred to | Text |  |
| Dt_discharge | Date of discharge, transfer or death | Date |  |
| Dt_last_test | Date of last COVID-19 test before discharge, if done | Date | Up to Sept. 14 |
| Result_last_test | Result of last COVID-19 test | Positive/Negative/Unknown | Up to Sept. 14 |
| Name_disch | Name of the person reporting the discharge^8^ | Text |  |
| Phone_disch | Phone number of the person reporting the discharge^8^ | Text |  |
| Email_disch | Email address of the person reporting the discharge^8^ | Text |  |
| ICU questionnaire (sub-questionnaire of discharge) | | | |
| Dt_reporting_ICU | Declaration date | Date |  |
| Hospital_name_ICU | Hospital name | Dropdown list |  |
| Dt_ICU_transfer | Date of transfer to ICU | Date |  |
| Length_stay_ICU | Length of stay at ICU | Numeric | Up to June 8 |
| QuickSOFA_score | QuickSOFA score at admission in ICU | Numeric | Up to June 8 |
| SOFA_score | SOFA (or pedSOFA > 18 years) score at admission in ICU: TOTAL - if sedated use for neurological SOFA the last available GCS on admission or before intubation | Numeric (total) |  |
| ICU_adm_reason | Reason for ICU admission | Dropdown list | From Sept. 14 |
| ICU_adm_reason_other | Reason for ICU admission, if other | Text | From Sept. 14 |
| Non_inv_vent_support | Non-invasive ventilatory support | Yes/No/Unknown |  |
| Length_non_inv_vent | Number of days on non-invasive ventilatory support | Numeric (days) | From Sept. 14 |
| Non_inv_vent_extub | Non-invasive ventilation only after extubation and weaning from invasive ventilatory support | Yes/No/Unknown | From Sept. 14 |
| Inv_Vent_support | Invasive ventilatory support | Yes/No/Unknown |  |
| Length_inv_vent | Number of days on invasive ventilatory support | Numeric (days) | From Sept. 14 |
| Weaning_inv_vent | Weaning of IVS | Yes (successfully weaned)/No(dead whilst on IVS)/Unknown | From Sept. 14 |
| ECMO | Oxigenation by ECMO | Yes/No/Unknown |  |
| Length_ECMO | Number of days on ECMO/ECCO2R | Numeric (days) | From Sept. 14 |
| Weaning_ECMO | Weaning of ECMO/ECCO2R | Yes (successfully weaned)/No(dead whilst on IVS)/Unknown | From Sept. 14 |
| Vasopressor | Vasopressor received? | Yes/No/Unknown | From Sept. 14 |
| Inotropic | Inotropic agent received? | Yes/No/Unknown | From Sept. 14 |
| Prone_posit | Prone positioning? | Yes/No/Unknown | From Sept. 14 |
| Renal_replace | Renal replacement therapy? | Yes/No/Unknown | From Sept. 14 |
| Tracheostomy | Tracheostomy? | Yes/No/Unknown | From Sept. 14 |
|  |  |  |  |
| Value_lactate_USI | Value of lactate at ICU admission | Numeric |  |
| Value_PaO2_FiO2_ratio_ICU | Lowest PaO2 / FiO2 on ICU admission (<=48h) | Numeric | From Sept. 14 |
| Value_CRP_USI | CRP value known at ICU admission? | Numeric |  |
| Value_Ddimer_ICU | Value of ddimeres on ICU admission | Numeric | From Sept. 14 |
| Value_ferritin_ICU | Value of ferritin on ICU admission | Numeric | From Sept. 14 |
| ICU_outcome | Patient was weaned from mechanical ventilation | Alive/Dead |  |
| Dt_ICU_discharge | Patient was weaned from ECMO | Date |  |
| ICU_dead_cause | If dead during ICU stay, reason | Dropdown list | From Sept. 14 |
| ICU_dead_other | Other reason for ICU death | Dropdown list | From Sept. 14 |
| Email_ICU | Email address of the person reporting the ICU^8^ | Text |  |

1. Date of inclusion (from …) / removal (up to …) of a variable in the questionnaire is only indicated if not collected from the initiation of the survey or if removed during the surveillance period up to 8^th^ of June (last survey update). [↑](#footnote-ref-1)
2. Questions asked only for patients below the age of 18 are indicated by (<18) [↑](#footnote-ref-2)
3. The official definition of 48 hours of hospitalization to consider an infection as nosocomial is not valid in the COVID-19 context, as SARS-CoV-2 has a longer incubation period. Thus, this reporting is based on the suspicion from the clinician, rather than the timing since hospital admission. [↑](#footnote-ref-3)
4. If the patient has been queried regarding the exposure, but no exposure was identified. [↑](#footnote-ref-4)
5. If the patient has not been queried regarding the exposure or there is no record of such an inquiry within the patient medical file. [↑](#footnote-ref-5)
6. Initially, it was not specified that cognitive conditions were excluded from the category “Comorbid_neuro_chronic”; from 23rd March on, the definition was clarified: “chronic neurologic conditions excluding cognitive disorders”, and the category “Cognitive disorders” was added. Cognitive disorder is indicated as “N/A” for the cases reported before the 23rd March. [↑](#footnote-ref-6)
7. Initially it was asked to report all patients with a laboratory-confirmed diagnosis of COVID-19. From the 3rd April on, cases confirmed with CT-scan were also recorded (in line with the change of the official case definition by Sciensano). Thus, before the 3rd of April, the method of diagnosis was not asked and the date of laboratory confirmation was recorded as dt_lab_confirmation. From the 3rd April on, the method of diagnostic confirmation is now asked as: diag_method_PCR / diag_method_scan / diag_method_age_rapid / diag_method_unk and the date of diagnosis is recorded as dt_diagnosis (if it is confirmed by several methods, the date of the first confirmation is asked). [↑](#footnote-ref-7)
8. Coordinates of the data providers are used only to recontact in case of question or communication related to the data but are not analyzed. [↑](#footnote-ref-8)
9. Includes pneumonia observed at the CT-scan and/or at the thoracic X-ray. [↑](#footnote-ref-9)
10. When patients are sufficiently well to be discharged from the hospital, but need to continue their recovery/convalescence at home or at another place outside the hospital.  [↑](#footnote-ref-10)
