# Supplementary material for: Rapid establishment of a national surveillance of COVID-19 hospitalizations in Belgium
Source: Arch Public Health. 2020 Nov 18;78:121. doi: 10.1186/s13690-020-00505-z (PMC7673251; doi:10.1186/s13690-020-00505-z)
Supplement: Supplementary file 3 — Additional file 3. Comparison baseline characteristics of patients in the Clinical survey based on the availability of admissions and/or discharge form. Baseline characteristics of patients in the Clinical survey with both discharge and admission forms vs patients without discharge form, and patients without admission form. [file 13690_2020_505_MOESM3_ESM.pdf]

Additional file 3: Baseline characteristics of patients in the Clinical survey with both discharge and admission forms vs patients without discharge form, and patients without admission form.

	**Patients with both forms(N=12891)**	**Patients without discharge form (N=2621)**	**p-value* no discharge vs both**	**Patients without admission form (N=1513)**	**p-value* no admission vs both**
**Gender** (n; % male)	6740/12749 (52.9)	1347/2620 (51.4)	0.17	819 (54.1)	0.35
**Age**, in years (mean; SD)	67.2 (18.6)	68.5 (19.0)	**<0.001**	67.9 (19.0)	0.14
**Comorbidities (n;%)**					
Hypertension	5052 (39.2)	1098 (41.9)	**0.01**	NA	
Cardiovascular disease	4372 (33.9)	897 (34.2)	0.76	NA	
Diabetes	2768 (21.5)	568 (21.7)	0.82	NA	
Chronic lung disease	1896 (14.7)	384 (14.7)	0.94	NA	
Chronic renal disease	1674 (13.0)	318 (12.1)	0.23	NA	
Cognitive disorder	1478/12167 (12.1)	314/2455 (12.8)	0.24	NA	
Obesity	857/8773 (9.8)	166/1770 (9.4)	0.35	NA	
Chronic neurological disease	1135 (8.8)	216 (8.2)	0.35	NA	
Solid cancer	1106 (8.6)	238 (9.1)	0.41	NA	
Chronic liver disease	341 (2.7)	75 (2.9)	0.53	NA	
Immunodepression	323 (2.5)	58 (2.2)	0.38	NA	
Haemotological cancer	234 (1.8)	56 (2.1)	0.27	NA	
None	2797 (21.7)	536 (20.5)	0.16		
**Admission to ICU** (n;%)	1743 (13.5)	NA		230 (15.2)	**0.02**
**Hospital outcome** (n; % death)	2627/12619 (20.8)	NA		378/1481 (25.5)	**<0.001**

Denominator indicated if not equal to total number of observations
** p-value: chi-square test for comparison of proportions and T-test for comparison of means (statistically significant are indicated in boldface)*

NA = not available for this subgroup of patients
