# Supplementary material for: Rapid establishment of a national surveillance of COVID-19 hospitalizations in Belgium
Source: Arch Public Health. 2020 Nov 18;78:121. doi: 10.1186/s13690-020-00505-z (PMC7673251; doi:10.1186/s13690-020-00505-z)
Supplement: Supplementary file 4 — Additional file 4. Comparison of mortality figures from the Clinical survey with the Surge Capacity survey. Comparison of the proportion of lab-confirmed deaths reported and the age and sex of lab-confirmed deceased persons between the Clinical survey and the Surge Capacity survey. [file 13690_2020_505_MOESM4_ESM.pdf]

Additional file 4: Comparison of mortality figures from the Clinical survey with the Surge Capacity survey.

	**Clinical survey**	**Surge Capacity survey**	**p-value***
**Proportion of deaths,** %	2373/11324[¥] (21.0)	4198/21178[§] (19.8)	**0.015**
**Gender of deceased persons,** % male	1327/2341 (56.7)	2325/3997[£] (58.2)	0.250
**Age of deceased persons**, mean (sd)	79.69 (10.48)	79.99 (11.03)[£]	0.274

[¥]Total number of lab-confirmed deaths as reported through the Clinical survey up to the 28th of June 2020 in the nominator and the total number of lab-confirmed patients with discharge information and discharged (alive or death) up to the 28th of June excluding the readmissions and transfers in the denominator.

[§]Total number of lab-confirmed deaths as reported through the Surge Capacity survey up to the 28th of June in the nominator and the sum of the number of lab-confirmed discharges (alive) and lab-confirmed deaths up to the 28th of June in the denominator.

[£]Individual data for each death (such as age and gender) is collected through the Surge Capacity survey from the 24th of March onwards.

*p-value: chi-square test for comparison of proportions and t-test for comparison of means (statistically significant are indicated in boldface)
